# Unseen Costs: The Direct and Indirect Impact of U.S. Immigration Policies on Child and Adolescent Health and Well‐Being

**DOI:** 10.1002/jts.22576

**Published:** 2020-08-13

**Authors:** T. Joseph Mattingly, Laurel Kiser, Sherika Hill, Ernestine C. Briggs, Carrie Purbeck Trunzo, Zafar Zafari, Theresa S. Betancourt

**Affiliations:** ^1^ Department of Pharmaceutical Health Services Research University of Maryland School of Pharmacy Baltimore Maryland USA; ^2^ Department of Psychiatry University of Maryland School of Medicine Baltimore Maryland USA; ^3^ UCLA/Duke University National Center for Child Traumatic Stress Department of Psychiatry and Behavioral Sciences Duke University Medical Center Durham North Carolina USA; ^4^ School of Social Work, Boston College Boston Massachusetts USA

## Abstract

Shifts in migration and border control policies may increase the likelihood of trauma exposure related to child–parent separation and result in costs to the health system and society. In the present study, we estimated direct and indirect costs per child as well as overall cohort costs of border control policies on migrant children and adolescents who were separated from their parents, detained, and placed in the custody of the United States following the implementation of the 2018 Zero Tolerance Policy. Economic modeling techniques, including a Markov process and Monte Carlo simulation, based on data from the National Child Traumatic Stress Network's Core Data Set (*N* = 458 migrant youth) and published studies were used to estimate economic costs associated with three immigration policies: No Detention, Family Detention, and Zero Tolerance. Clinical evaluation data on mental health symptoms and disorders were used to estimate the initial health state and risks associated with additional trauma exposure for each scenario. The total direct and indirect costs per child were conservatively estimated at $33,008, $33,790, and $34,544 after 5 years for No Detention, Family Detention, and Zero Tolerance, respectively. From a health system perspective, annual estimated spending increases ranged from $1.5 million to $14.9 million for Family Detention and $2.8 million to $29.3 million for Zero Tolerance compared to baseline spending under the No Detention scenario. Border control policies that increase the likelihood of child and adolescent trauma exposure are not only morally troubling but may also create additional economic concerns in the form of direct health care costs and indirect societal costs.

In 2014, the United States faced an influx of migrants, a term used herein to encompass all individuals attempting to cross into the United States, including those who meet the legal definition of refugee or those seeking asylum, at the southern border; this was characterized as a “surge,” with families seeking asylum from Central America (Eagly et al., 2018; Lind, 2014). This resulted in a significant increase in family detention rather than a release of individuals with notices to appear in immigration court at a later date (Eagly et al., 2018). In April 2018, the U.S. Justice Department began prosecuting 100% of individuals or families apprehended by the border patrol, which resulted in the separation of a reported 2,342 children from parents who were suspected of illegal entry (Lind, 2018a; Rizzo, 2018; U.S. Department of Homeland Security, 2018). Recently, court filings claiming that detention center conditions did not meet safe and sanitary requirements led to a district judge ordering the U.S. Customs and Border Protection agency to permit clinicians into two facilities, El Paso and Rio Grande Valley, in Texas (McLaughlin & Moshtaghian, 2019). Additionally, video of a U.S. Justice Department lawyer during a Ninth Circuit Court of Appeals hearing sparked outrage as it appeared that the government was arguing that toothbrushes, soap, towels, showers, blankets, and adequate sleeping conditions were not requirements for temporary detention standards (Fernandez, 2019; Flynn, 2019a). The political debate over access to living conditions has garnered considerable media attention and has raised the pressing health‐related question: What are the direct and indirect health‐related costs of these shifting immigration policies given the risk for traumatic separation and loss among children?

Children exposed to traumatic experiences are at an increased risk of a range of detrimental health outcomes, including posttraumatic stress disorder (PTSD), anxiety, depression, sleep disorders, exacerbated attention‐deficit/hyperactivity disorder (ADHD) and asthma symptomology, and somatic complaints and distress (Betancourt et al., 2012; Jimenez et al., 2017; Oh et al., 2018). These risks are heightened for migrant children who have experienced trauma within their family and in their country of origin (Betancourt et al., 2012; Humphreys, 2019). Moreover, experts have argued that actions that “unnecessarily and traumatically” remove children from their parents can have detrimental effects on physical and mental health, particularly for those who have been exposed to trauma before or during migration (Humphreys, 2019; MacKenzie et al., 2017). Recently, the findings from a cross‐sectional study of migrant children held at detention centers demonstrated that children forcibly separated from their mothers had elevated scores on measures of emotional problems, peer problems, and total difficulties, providing evidence that forced separation may be associated with mental health distress (MacLean et al., 2019).

Betancourt et al. (2017) utilized the National Child Traumatic Stress Network (NCTSN) Core Data Set (CDS) to compare a sample of refugee children to propensity‐matched cohorts of immigrants and U.S.‐born children. In this analysis, both refugees and immigrants endorsed significantly higher ratings of day‐to‐day functional impairment and a higher number of trauma types compared with U.S.‐origin youth. For instance, refugees had higher levels of several clinical disorders than the propensity‐matched U.S. youth, specifically regarding traumatic complicated grief, dissociation, somatization, sexual behavior problems, oppositional defiant disorder, substance abuse, and phobic disorder, which also exceeded the rate in the matched immigrant group. In addition, analyses demonstrated a dose–response relationship whereby the endorsement of a higher number of traumatic experiences was associated with more severe functional impairment and a higher level of need for and utilization of services (Betancourt et al., 2017).

The present study expanded on the research by Betancourt and colleagues by utilizing the NCTSN–CDS to develop a decision‐analytic framework to investigate the potential impact of separating all children and adolescents from parents (i.e., Zero Tolerance) compared with previous policies to detain families together (i.e., Family Detention) or release families with a notice to appear in court at a later date (i.e., No Detention). Family unity has been an important part of U.S. migration policies since the 1960s, but the process and implementation of these policies have changed dramatically, with more formal detention expanding in the early 2000s to include up to five brick‐and‐mortar detention centers (Eagly et al., 2018; Gubernskaya & Dreby, 2017). In 2014, an increase in migration from Central America led to additional temporary detention camps and an increase in detention center bed capacity to house children and adolescents with their parents (Eagly et al., 2018). The determination to prosecute 100% of adult migrants was referred to as the “Zero Tolerance” policy and resulted in forced separation in cases in which the adults were accompanied by minors (Humphreys, 2019; U.S. Department of Homeland Security, 2018). To our knowledge, there has not been an economic analysis of the direct and indirect health‐related costs of immigration policies related to the health consequences of childhood trauma exposure. Accordingly, the present project aimed to provide a conservative cost estimate of the 2018 Zero Tolerance policy in comparison to previous policies.

## Method

### Participants

The present study used a subsample of migrant children and adolescents between 3 and 20 years of age from the NCTSN–CDS. The NCTSN is a federally funded child mental health service initiative aimed at improving the standard of care for children who have been exposed to trauma in the United States (Pynoos et al., 2008). The CDS was collected between 2004 and 2012 and captured data on youth who accessed treatment services from 74 NCTSN centers around the United States. The CDS includes comprehensive information on demographic characteristics, functional impairment, clinical diagnoses, and trauma history details, along with information on treatment services for youth from birth to 21 years of age. Clinician assessments were used to generate the Clinical Evaluation data, which includes ratings of the degree to which clinical problems, symptoms, or disorders were displayed by the child at baseline. Ratings were made on a 3‐point scale consisting of 0 (*not present*), 1 (*possibly present*), and 2 (*definitely present*). Clinicians also used patient and other informant reports to complete the Indicators of Severity of Problems (IOS) form, which assessed the degree of functional impairment across a broad range of psychosocial domains and contexts, including home, school, and community. The 14 IOS items represent day‐to‐day functional impairments commonly observed in trauma‐exposed patients, including mental health needs (Betancourt et al., 2017); high‐risk health behaviors, such as substance use (Layne, Greeson, et al., 2014); consequences related to risk factors, including problems at home or school (Layne, Briggs, et al. 2014); and histories of mental health service use (Briggs et al., 2013). We divided the sample into quartiles based on the number of IOS items endorsed to create a distribution of ordinal ranking. To ensure consistency with published literature on adverse childhood experiences showing the additive impact of each additional traumatic experience (i.e., trauma count) on psychopathology (Putnam et al., 2013), we used IOS quartiles as a categorical, ordinal variable. Quartile 1 included individuals who endorsed zero IOS items, Quartile 2 represented one or two IOS items, Quartile 3 represented three or four IOS items, and Quartile 4 represented more than four endorsed IOS items.

The trauma history portion of the CDS was derived from the NCTSN–CDS General Trauma History and Trauma Details forms as well as items from the UCLA PTSD Reaction Index (Steinberg et al., 2004). Information about trauma history, traumatic loss, bereavement, and separation were obtained from multiple informants, including the child or adolescent, parents or caregivers, and other relatives or collaterals. The General Trauma form included a comprehensive list of 20 types of trauma, loss, and separation exposures. Trained providers endorsed whether the child experienced each type of trauma or whether the trauma type was suspected to have occurred based on the child, caregiver, or collateral reports. For the present study, only confirmed occurrences of trauma exposure were included. All procedures were approved by the Duke University Health System Institutional Review Board and the respective institutional review boards (IRBs) of all participating NCTSN sites. The University of Maryland IRB determined the economic analysis used in this study was not human subject research.

A subsample (*N* = 458) of migrant children and adolescents from the CDS was used in the present study. To closely reflect the present‐day population of migrants at the United States–Mexico border, the CDS subsample was identified based on three criteria: a Latin American country of origin; (b) self‐report as a refugee, immigrant, or asylee; and (c) endorsement of experiencing war, terrorism, or political victimization outside of the United States. The CDS cohort was used to inform simulation model assumptions applied to a hypothetical cohort of migrant children and adolescents impacted by 2018 policy changes (U.S. Department of Homeland Security, 2018).

### Procedure

**Figure 1:**
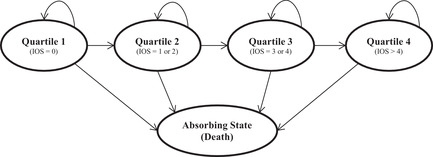
Markov Process With Advancing Progression, by Number of Indicators of Severity (IOS) Items

A health‐state transition model was used to estimate the potential costs of PTSD, anxiety, depression, and ADHD from health‐sector and societal perspectives over 5‐ and 10‐year periods. A short‐term time horizon was chosen to limit extending our assumptions throughout a lifetime model given the implications of other economic factors not measured herein, such as inflation and other market‐driven factors, such as health care reform, that could greatly influence cost fluctuations. In addition, research has shown that the median time for seeking mental health treatment after the onset of symptoms is 4 years (Wang et al., 2007). Accordingly, the 5‐ and 10‐year models attempt to capture the potential delay or failure of seeking treatment that would not be obvious in a 1‐year model that follows the first year after symptom onset. Different states (i.e., quartiles) were defined by the frequency distribution of IOS items that capture functional impairments commonly observed in youth who have experienced trauma (Figure 1). An age‐based annual mortality estimate was used for each 1‐year cycle without assigning additional mortality risk to higher IOS quartiles. A cumulative logit model was conducted in SAS (Version 9.4) to estimate the odds of increasing to the next quartile level for each additional trauma exposure. The IOS quartile was the dependent variable, and the number of traumatic experiences was the independent variable.

Policy scenarios were modeled to represent three different types of actions taken by the U.S. government in response to family migration. These scenarios included No Detention, Family Detention, and Zero Tolerance. No Detention refers to the period before 2014 during which border patrol agents who apprehended migrants crossing the United States–Mexico border would open up a case in court and release the migrant, who was expected to show up for a specified court date (Lind, 2018b). Family Detention refers to the period from 2014 to April 2018 when families caught at the border were detained together for a period of fewer than 20 days (Lind, 2018b). Zero Tolerance refers to the post–April 2018 period during which 100% of detained adult migrants traveling across the United States–Mexico border were prosecuted, effectively forcing the separation of any children accompanying the adult accused of illegal entry (U.S. Department of Homeland Security, 2018).

### Measures

#### Clinical Evaluation

Clinical inputs included the prevalence of PTSD, anxiety, depression, and ADHD in each of the four IOS quartiles, estimated from the CDS cohort (Supplemental Table S1). The current study used diagnostic criteria from the fourth edition of the *Diagnostic and Statistical Manual of Mental Disorders* (*DSM‐IV*), rating disorders as “definitely present” to establish conservative estimates for the prevalence of depression, anxiety, and ADHD. The prevalence of PTSD was derived from either a clinically significant score on the UCLA PTSD Reaction Index (Steinberg et al., 2004) or a designation of “definitely present” on the Clinical Evaluation form if PTSD standardized scores were not available. The initial health‐state probabilities for the No Detention scenario corresponded to the four IOS quartiles derived from the distribution found in the CDS, given that the assumption that these youth were not exposed to additional traumatic experiences due to government policy, as the families were released as a unit into the broader community. The initial health‐state probabilities for Family Detention included an assumption that 50% of the children in the Family Detention group would experience an additional policy‐related trauma while being detained with family members, which could include illness or death of a family member, witness to community violence, or other traumatic experiences such as bullying, medical trauma, neglect, abuse, domestic violence, or an impaired caregiver. To be consistent with data on U.S. youth, 50% is a justifiable assumption given that national data show that 54% of youth report experiencing a traumatic event 18 years of age (Child Trends, 2018). This assumption was further tested using a one‐way sensitivity analysis. In the Zero Tolerance group, 100% of children would be exposed to an additional policy‐related trauma due to separation from parents or guardians, which can cause traumatic grief, as well as refugee trauma related to displacement (National Child Traumatic Stress Network, 2020). We assumed the annual rate of new trauma exposure unrelated to immigration would be equal for all three groups (Finkelhor, Turner, Shattuck, & Hamby, 2015).

#### Health‐Related Costs

The odds of starting in a more severe IOS health‐state after experiencing trauma were calculated using an ordinal logistic regression model from the CDS cohort, which was adjusted for age and gender (male or female). Annual health‐state transition probabilities, direct costs for health‐sector expenditures, and indirect costs for societal estimates (e.g., time costs, caregiver absenteeism, transportation) for each IOS health‐state were derived from the literature, and all costs were converted to 2018 U.S. dollars (USD; Supplemental Table S1). Direct and indirect cost estimates were selected for PTSD, anxiety, depression, and ADHD based on the availability of evidence. Children who occupied the least‐severe state were assigned annual costs associated with no behavior problems (Hakkaart‐van Roijen et al., 2007). With health‐state progression, costs associated with behavior problems were included along with additional costs associated with PTSD, anxiety, depression, and ADHD, by multiplying the probabilities that children occupying that health‐state would experience those clinical diagnoses (Bodden et al., 2008, 2018; Hakkaart‐van Roijen et al., 2007).

### Data Analysis

The base‐case analysis included costs per child for both health‐sector and societal perspectives. The confidence interval for the primary cost‐per‐child outcome in the model was based on the lower and upper bounds of the 95% confidence interval in the CDS subsample's adjusted regression model on the effects of additional (i.e., incremental) trauma exposures. The cost‐per‐child estimate was applied to three hypothetical child cohorts for the total health sector and societal costs of each policy scenario (Lind, 2018a). The first cohort included an estimated 2,342 children separated from parents reported on June 19, 2018, as a result of the Zero Tolerance policy change (Lind, 2018a). The second (*n* = 16,790) and third (*n* = 23,725) cohorts were annual estimates calculated by a recent news report of 46–65 daily parent‐child separations (Lind, 2018a). This analysis gives an approximation of the total annual spending needed to meet the health care needs of these cohorts, assuming a continuous application of each border control policy.

One‐way deterministic sensitivity analyses were conducted by changing input parameters across a conservative high–low range for each input listed in Supplemental Table S1 (Caro et al., 2012). The assumption regarding the impact of trauma exposure attributed to the policy change was further tested in a one‐way sensitivity analysis in which we increased the proportion of children who experienced an additional trauma exposure from 0% to 100%, in increments of 10%. The most sensitive input parameters were included in a tornado diagram for both health‐sector and societal perspectives. A 10‐year model was also conducted and reported for both perspectives.

## Results

The final analytic sample consisted of 458 migrant children and adolescents within the NCTSN–CDS who had complete information for the IOS form and Clinical Evaluation items regarding PTSD, generalized anxiety disorder, depression, and ADHD. Baseline sample demographics are reported in Table 1. Of the 458 children observed in the CDS, 13.1% did not endorse any IOS items (Quartile 1), 38.2% endorsed one or two items (Quartile 2), 29.3% endorsed three or four items, and 19.5% endorsed more than four IOS items at baseline (Table 2). The odds of increasing to a higher IOS quartile with each additional trauma type were 1.232, 95% CI [1.141, 1.330], adjusted for age and sex.

**Table 1:** Baseline Core Data Set (CDS) Sample Demographic Characteristics of Immigrant Children From Relevant Countries

Characteristic	*M*	*SD*	*n*	%
Age (years)	13	4		
Gender				
Male			197	43.0
Female			261	57.0
Ethnicity				
Hispanic or Latino			441	96.3
Non‐Hispanic or Latino			17	3.7
Current legal guardian				
Parent			341	74.4
Other relative			34	7.4
Other (state, unknown, missing)			83	18.1
Other unknown				
Insurance				
None			236	51.5
Public			133	29.0
Private			43	9.4
Other (unknown, missing)			46	10.0
Number of traumatic events experienced	3.6	2.3		

*Note. N* = 458.

**Table 2:** Baseline Prevalence of Disease, by Indicator of Severity (IOS) Frequency

	Full sample (*N* = 458)		No IOS (*n* = 60)		1–2 IOS (*n* = 175)		3–4 IOS (*n* = 134)		>4 IOS (*n* = 89)	
Variable	*n*	%	*n*	%	*n*	%	*n*	%	*n*	%
PTSD	195	42.58	21	35.00	60	34.29	70	52.24	44	49.44
Depression	109	23.80	7	11.67	32	18.29	32	25.37	36	40.45
Anxiety	39	8.52	4	6.67	12	6.86	15	11.19	8	8.99
ADHD	10	2.18	1	1.67	2	1.14	6	4.48	1	1.12

*Note*. ADHD = attention‐deficit hyperactivity disorder; PTSD = posttraumatic stress disorder.

### Model Results

In the 5‐year scenario, the total direct costs per child were $23,652, $24,281, and $24,887 for No Detention, Family Detention, and Zero Tolerance, respectively. The incremental health‐ sector costs per child of Family Detention and Zero Tolerance were $629 and $1,235, respectively, when compared with the baseline No Detention policy. When the indirect costs were included in the societal perspective model, total costs were $33,008 per child for No Detention, $33,790 per child for Family Detention, and $34,544 per child for Zero Tolerance. The resulting incremental costs, including both direct and indirect costs, of Family Detention and Zero Tolerance were $782 and $1,536, respectively, when compared with the baseline No Detention policy (Table 3).

**Table 3:** Base Per‐Child Health Sector and Societal Cost Estimates and Incremental Costs Applied to Hypothetical Cohorts in 5‐ and 10‐Year Models

Variable	No Detention	Family Detention	Zero Tolerance
5‐year model			
Total direct costs per child	$23,652^a^	$24,281	$24,887
Incremental costs for Cohort 1	‐	$1,473,118	$2,892,370
Incremental costs for Cohort 2	‐	$10,560,910	$20,735,650
Incremental costs for Cohort 3	‐	$14,923,025	$29,300,375
Total direct and indirect costs per child	$33,008^a^	$33,790	$34,544
Incremental costs for Cohort 1	‐	$1,831,440	$3,597,312
Incremental costs for Cohort 2	‐	$13,129,780	$25,789,440
Incremental costs for Cohort 3	‐	$18,552,950	$36,441,600
10 ‐year model			
Total direct costs per child	$49,593^a^	$50,734	$51,875
Incremental costs for Cohort 1	‐	$2,672,222	$5,344,444
Incremental costs for Cohort 2	‐	$19,157,390	$38,314,780
Incremental costs for Cohort 3	‐	$27,070,225	$54,140,450
Total direct and indirect costs per child	$74,139^a^	$75,644	$77,158
Incremental costs for Cohort 1	‐	$3,524,710	$7,070,498
Incremental costs for Cohort 2	‐	$25,268,950	$50,689,010
Incremental costs for Cohort 3	‐	$35,706,125	$71,625,775

*Note*. Cohort 1: *n* = 2,343; Cohort 2: *n* = 16,790; Cohort 3: *n* = 23,725.

a Reference category.

From a health‐sector budgetary perspective, after applying the costs per child to the three hypothetical cohorts (*n* = 2342, *n* = 16790, and *n* = 23,725), project spending increases ranged from $1.5 million to $14.9 million for Family Detention and from $2.8 million to $29.3 million for Zero Tolerance, compared to the baseline spending under the No Detention scenario after 5 years. The estimated additional spending, including indirect costs, ranged from $1.8 million to $18.6 million for Family Detention and $3.6 million to $36.4 million for Zero Tolerance, compared to the baseline spending under No Detention (Table 3).

### Sensitivity Analysis

The results of one‐way sensitivity analyses demonstrated that both health‐sector and societal models were most sensitive to the direct health‐cost estimates related to depression. For example, as cost inputs attributable to depression were adjusted in one‐way analyses, the total costs varied from approximately $19,000 to $28,000 per child in the health sector model. All variables that had an impact on the model results are reported in the Supplementary Materials. For the policy‐related trauma assumption, a 10% increase in the proportion of children and adolescents increased the incremental costs per child by $126 in the health‐sector model and by $168 in the societal model (Supplemental Table S2). When the model was extended to 10years, the estimated additional spending increased for the Family Detention and Zero Tolerance scenarios compared to baseline spending (Table 3).

## Discussion

The present analysis was the first to model the potential economic impact of child–parent separation and family detention policies for present‐day Hispanic migrants at the southern border of the United States. We combined social and emotional well‐being and functioning in a clinically referred sample of migrant children and published literature to estimate how systematically exposing children to additional trauma may increase the direct and indirect health care expenditures over a 5‐ and 10‐year periods. In addition to the clinical, humanitarian, and ethical concerns that have been raised (MacKenzie et al., 2017), we demonstrate that there are substantial economic costs for U.S. government border‐enforcement policies with regard to child trauma exposure, especially given the existing health state of migrant populations. This modeling approach can help inform policymakers by raising awareness of potential direct and indirect costs that may occur downstream as a result of the enforcement of policies that expose children and adolescents to additional trauma.

The CDS cohort utilized in the present study was consistent with observations of mental health and behavioral issues of traumatized migrant youth reported by MacLean et al. (2019) regarding children forcibly separated from their mothers at the U.S. border. In both samples, nearly half of the children endorsed high levels of symptom ratings on the UCLA PTSD Reaction Index (MacLean et al., 2019). In contrast, Betancourt et al. (2017) found increased rates of depression, anxiety, and ADHD in their sample when comparing refugee, immigrant, and U.S.‐born children who were propensity score‐matched on at least one confirmed trauma type. The present study did not compare migrants to U.S.‐born children but rather used the baseline estimates from children in the data that closely reflect the present‐day population of migrants at the United States–Mexico border. Stratification of our CDS sample based on groupings of IOS items from none, one to two, three to four, and more than four offers both conservative estimates of prevalence rates and increased granularity of the comorbidity between functional impairment and psychiatric diagnoses of PTSD, anxiety, depression, and ADHD.

There are several limitations to estimating the potential future health‐sector and societal costs through assumptions of additional trauma exposure added by current policies without direct observation of or primary data for the exact population. First, the total number of children and adolescents impacted by these policies are assumptions based on journalist reports that are not confirmed by a government source. Second, the trauma construct in this model represents a quantitative variable without a degree or level of severity. Although recent news reports have described difficult detention center conditions, with children being detained without adequate food, water, and sanitation, we have no primary data on the potential policy‐related trauma exposure for children in each scenario (Flynn, 2019b; Lithwick & Schlanger, 2019). However, we did empirically test the association between endorsement of one additional trauma type and the likelihood a child would transition to the next IOS quartile after controlling for differences in sex and race. We observed a 23% increased chance an individual would advance to a higher state in our available sample of 458 children and adolescents. A larger sample or actual data from this specific cohort of youth who migrated after April 2018 would improve this input assumption. By estimating the risk of advanced IOS quartiles from cross‐sectional data collected on a similar group of children who migrated prior to the child–parent separation policy, we may be underestimating the level of trauma inflicted as well as the incidence of anxiety, depression, PTSD, and ADHD that may eventually be observed by children who potentially experience more difficult environments compared to their 2004–2012 counterparts. Nonetheless, we acknowledge that the CDS cohort, which consisted of youth who “successfully” migrated to the United States, could be statistically different from present‐day youth attempting to migrate with regard to numerous characteristics not tested in the model herein.

Other potential health consequences of childhood trauma exposure include attempted suicide, illicit drug use, alcoholism, sleep disorders, sexually transmitted diseases, and overall mortality (Bellis et al., 2015; Betancourt et al., 2012, 2017; Dube et al., 2003). Since 2004, approximately 97 migrants have died in detention, with at least seven children dying in custody in the past year (Pompa, 2019). We were unable to find high‐quality economic literature to support including costs associated with these other potential health consequences for our population. When accounting for these other potential health risks and possible loss of life, the total direct and indirect costs could be substantially higher.

With regard to costs, our direct and indirect cost estimates were derived from studies in non–U.S. settings. Although we converted all costs to U.S. dollars, this potentially underestimates actual spending on these services in the United States, as many health services are often more expensive in the United States compared to other countries. Moreover, we assessed the frequencies of mental health outcomes using a sample of youth aged 3 years and older. Arguably, the inclusion of the additional costs for youth who experienced trauma in the first three years of life would yield higher cost estimates.

In addition, our analysis did not include the costs of detention or other non–health‐related costs to the U.S. government as a result of the policy changes. Both the Family Detention and Zero Tolerance policies increase the number of individuals detained and housed by the U.S. government, which has been estimated at $126 per person per day (Eagly et al., 2018). Considering the increased expenditures from direct and indirect health costs in our model, the additional non–health‐related costs of implementing Family Detention and Zero Tolerance further signifies the cost–benefit in favor of No Detention. Furthermore, this model represents a static policy environment and does not account for the frequency of policy changes or facility‐level implementation of the existing policies. Future research may consider the costs spent on government detention in comparison with potential efforts by non‐profit organizations, such as foundations, churches, or humanitarian shelters, whereby asylum seekers may be provided with different levels of care or support until they are scheduled for their court appointments. In addition, the limitations in this modeling approach demonstrate a need for future research that directly observes this population over time to better understand the lived experiences, health outcomes, and economic burdens faced by these families.

The present study provides a conservative estimate of the total and incremental costs related to health consequences that are potentially attributable to immigration policy changes. Sensitivity analyses were conducted to provide a range of outputs based on different input assumptions. In addition, two short‐term forecast models of 5 and 10 years were constructed to demonstrate the potential impacts in the short term without accounting for market fluctuations that could increase health care pricing in the long term. Collectively, these results show that border control policies that increase the likelihood children will experience trauma may create additional economic concerns in the form of direct health care costs and indirect societal costs. Modeling costs using expert opinion, peer‐reviewed literature, and estimates from a real‐world sample of comparable children and adolescents provides a first step to understanding the implications of policy decisions. However, empirical evidence that directly evaluates the children and adolescents who are actually exposed to the most recent policies would help confirm or improve our modeling assumptions.

## Supporting information


***Table S1***: *Reference model inputs and assumption variation for deterministic sensitivity analyses*.
***Table S2***: *Impact of additional policy‐related trauma assumption on incremental costs per child at 5 and 10 years*.
***Figure S1*** : *Deterministic sensitivity analysis for health sector perspective*.
**Figure S2**: Deterministic sensitivity analysis for societal perspective.
