# Supplementary material for: Unseen Costs: The Direct and Indirect Impact of U.S. Immigration Policies on Child and Adolescent Health and Well‐Being
Source: J Trauma Stress. 2020 Aug 13;33(6):873–81. doi: 10.1002/jts.22576 (PMC7754407; doi:10.1002/jts.22576)
Supplement: Supplementary file 1 — Table S1: Reference model inputs and assumption variation for deterministic sensitivity analyses. Table S2: Impact of additional policy‐related trauma assumption on incremental costs per child at 5 and 10 years. Figure S1 : Deterministic sensitivity analysis for health sector perspective. Figure S2: Deterministic sensitivity analysis for societal perspective. [file JTS-33-873-s001.docx]

***Table S1:*** *Reference model inputs and assumption variation for deterministic sensitivity analyses.*

| **Input** | **Base Case** | **Range** | **Source** |
| --- | --- | --- | --- |
| Age at Entry | 13.0 | 5.0-15.0 | NCTSN |
| Discount Rate | 0.03 | 0.01-0.05 | (Caro, Briggs, Siebert, Kuntz, & ISPOR-SMDM Modeling Good Research Practices Task Force, 2012) |
|  |  |  |  |
| **Transition Probabilities** |  |  |  |
| Adjusted Odds of Higher IOS with Additional Trauma | 1.232 | 1.141-1.330 | NCTSN |
| Annual Progression Rate to Higher Quartile | 0.1 | 0.0-0.15 | Estimate |
| Annual Regression Rate to Lower Quartile | 0.05 | 0.0-0.1 | Estimate |
|  |  |  |  |
| **Costs** |  |  |  |
| ADHD (Direct) | 2,725 | 0-2,725 | (Guevara, Lozano, Wickizer, Mell, & Gephart, 2001; Hakkaart-van Roijen et al., 2007) |
| ADHD (Indirect) | 2,781 | 0-2,781 | (Guevara et al., 2001; Hakkaart-van Roijen et al., 2007) |
| Anxiety (Direct) | 2,690 | 1,546-3,834 | (Bodden, Dirksen, & Bögels, 2008) |
| Anxiety (Indirect) | 2,343 | 1,415-3,271 | (Bodden et al., 2008) |
| Depression (Direct) | 12,443 | 8,745-16.141 | (Bodden, Stikkelbroek, & Dirksen, 2018) |
| Depression (Indirect) | 3,680 | 2,907-4,454 | (Bodden et al., 2018) |
| Behavior Problems (Direct) | 436 | 0-500 | (Hakkaart-van Roijen et al., 2007) |
| Behavior Problems (Indirect) | 765 | 0-1,000 | (Hakkaart-van Roijen et al., 2007) |
| PTSD (Direct) | 1,873 | 1,408-3,281 | (Shearer et al., 2018) |
| PTSD (Indirect) | 954 | 0-1,273 | (Salloum et al., 2016) |
| No issues – Baseline (Direct) | 332 | 0-500 | (Hakkaart-van Roijen et al., 2007; Swensen et al., 2003) |
| No issues – Baseline (Indirect) | 645 | 0-1,000 | (Hakkaart-van Roijen et al., 2007; Swensen et al., 2003) |
|  |  |  |  |
| **Model Prevalence Rates by Quartile** |  |  |  |
| ADHD_Q1 | 0.0167 | 0.01-0.03 | NCTSN |
| ADHD_Q2 | 0.0114 | 0.01-0.03 | NCTSN |
| ADHD_Q3 | 0.0448 | 0.03-0.05 | NCTSN |
| ADHD_Q4 | 0.0112 | 0.01-0.03 | NCTSN |
| Anxiety_Q1 | 0.0667 | 0.05-0.1 | NCTSN |
| Anxiety_Q2 | 0.0686 | 0.05-0.1 | NCTSN |
| Anxiety_Q3 | 0.1119 | 0.1-0.15 | NCTSN |
| Anxiety_Q4 | 0.0899 | 0.075-0.125 | NCTSN |
| Depression_Q1 | 0.1167 | 0.1-0.15 | NCTSN |
| Depression_Q2 | 0.1829 | 0.15-0.2 | NCTSN |
| Depression_Q3 | 0.2537 | 0.2-0.3 | NCTSN |
| Depression_Q4 | 0.4045 | 0.3-0.5 | NCTSN |
| PTSD_Q1 | 0.3500 | 0.3-0.5 | NCTSN |
| PTSD_Q2 | 0.3429 | 0.3-0.5 | NCTSN |
| PTSD_Q3 | 0.5224 | 0.4-0.6 | NCTSN |
| PTSD_Q4 | 0.4944 | 0.4-0.6 | NCTSN |
| Abbreviations: ADHD=attention-deficit hyperactivity disorder, IOS=Indicators of Severity, NCTSN=National Child Trauma Stress Network, PTSD=posttraumatic stress disorder, Q=Quartile | | | |

***Table S2:*** *Impact of additional policy-related trauma assumption on incremental costs per child at 5 and 10 years.*

|  | **Direct Costs Only** | **Direct and Indirect Costs** |
| --- | --- | --- |
| *Proportion of Children Where Policy Change Led to Trauma* | | |
| 0% (Base Case for “No Detention”) | $0 [Reference] | $0 [Reference] |
| 10% | $126.06 | $168.18 |
| 20% | $252.12 | $336.35 |
| 30% | $378.18 | $504.53 |
| 40% | $504.24 | $672.70 |
| 50% (Base Case for “Family Separation”) | $630.30 | $840.88 |
| 60% | $756.36 | $1,009.08 |
| 70% | $882.42 | $1,177.23 |
| 80% | $1,008.48 | $1,345.41 |
| 90% | $1,134.54 | $1,513.59 |
| 100% (Base Case for “Zero Tolerance”) | $1,260.60 | $1,681.76 |

***Figure S1****: Deterministic sensitivity analysis for health sector perspective.*


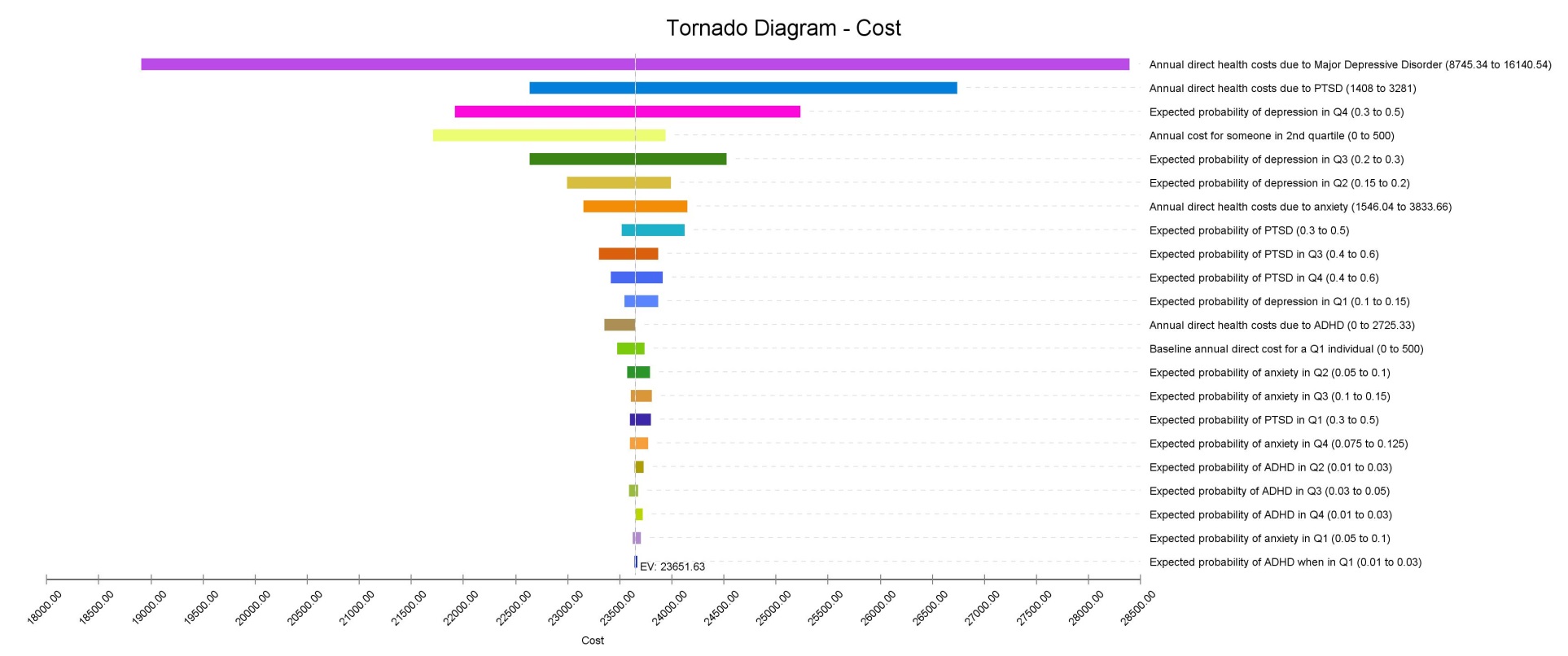


**Figure S2**: Deterministic sensitivity analysis for societal perspective.


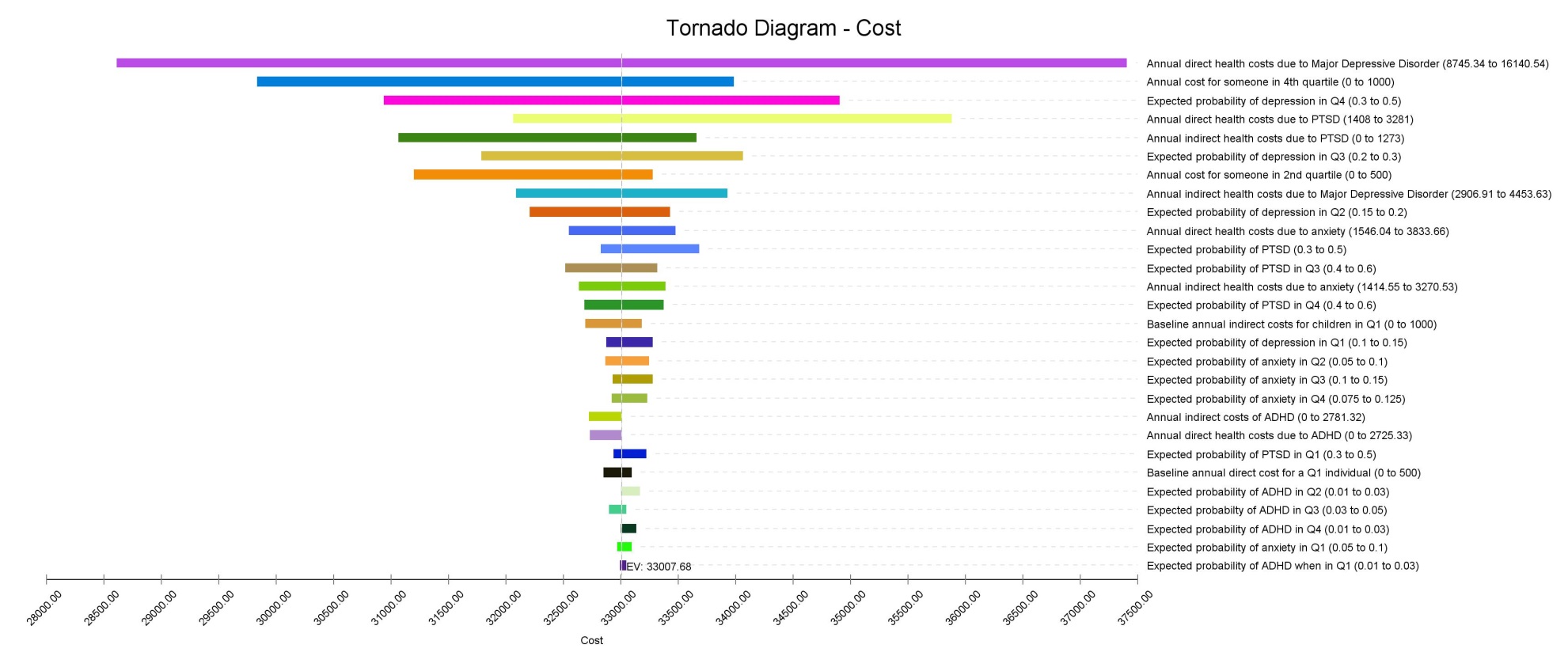


**References:**

Bodden, D. H. M., Dirksen, C. D., & Bögels, S. M. (2008). Societal burden of clinically anxious youth referred for treatment: A cost-of-illness study. *Journal of Abnormal Child Psychology*, *36*(4), 487–497. https://doi.org/10.1007/s10802-007-9194-4

Bodden, D. H. M., Stikkelbroek, Y., & Dirksen, C. D. (2018). Societal burden of adolescent depression, an overview and cost-of-illness study. *Journal of Affective Disorders*, *241*(May), 256–262. https://doi.org/10.1016/j.jad.2018.06.015

Caro, J. J., Briggs, A. H., Siebert, U., Kuntz, K. M., & ISPOR-SMDM Modeling Good Research Practices Task Force. (2012). Modeling good research practices--overview: A report of the ISPOR-SMDM Modeling Good Research Practices Task Force--1. *Medical Decision Making : An International Journal of the Society for Medical Decision Making*, *15*(6), 796–803. https://doi.org/10.1016/j.jval.2012.06.012

Guevara, J., Lozano, P., Wickizer, T., Mell, L., & Gephart, H. (2001). Utilization and cost of health care services for children with Attention-Deficit/Hyperactivity Disorder. *Pediatrics*, *108*(1), 71–78.

Hakkaart-van Roijen, L., Zwirs, B. W. C., Bouwmans, C., Tan, S. S., Schulpen, T. W. J., Vlasveld, L., & Buitelaar, J. K. (2007). Societal costs and quality of life of children suffering from attention deficient hyperactivity disorder (ADHD). *European Child and Adolescent Psychiatry*, *16*(5), 316–326. https://doi.org/http://dx.doi.org/10.1007/s00787-007-0603-6

Salloum, A., Wang, W., Robst, J., Murphy, T. K., Scheeringa, M. S., Cohen, J. A., & Storch, E. A. (2016). Stepped care versus standard trauma-focused cognitive behavioral therapy for young children. *Journal of Child Psychology and Psychiatry and Allied Disciplines*, *57*(5), 614–622. https://doi.org/10.1111/jcpp.12471

Shearer, J., Papanikolaou, N., Meiser-Stedman, R., McKinnon, A., Dalgleish, T., Smith, P., … Byford, S. (2018). Cost-effectiveness of cognitive therapy as an early intervention for post-traumatic stress disorder in children and adolescents: A trial based evaluation and model. *Journal of Child Psychology and Psychiatry and Allied Disciplines*, *59*(7), 773–780. https://doi.org/10.1111/jcpp.12851

Swensen, A. R., Birnbaum, H. G., Secnik, K., Marynchenko, M., Greenberg, P., & Claxton, A. (2003). Attention-deficit/hyperactivity disorder: Increased costs for patients and their families. *Journal of the American Academy of Child and Adolescent Psychiatry*, *42*(12), 1415–1423. https://doi.org/10.1097/00004583-200312000-00008
